# (5*S*)-4-(2,2-Dimethyl­prop­yl)-5-isopropyl-1,3,4-oxadiazinan-2-one

**DOI:** 10.1107/S1600536810048798

**Published:** 2010-11-27

**Authors:** Kate L. Edler, Sharon E. E. Kirk, Ryan A. Davis, Shawn R. Hitchcock, Gregory M. Ferrence

**Affiliations:** a CB 4160, Department of Chemistry, Illinois State University, Normal, IL 61790, USA

## Abstract

The title compound, C_11_H_22_N_2_O_2_, has one chiral center and packs in the monoclinic space group *P*2_1_. The asymmetric unit has five crystallographically independent mol­ecules, four of which engage in inter­molecular N—H⋯O hydrogen bonding.

## Related literature

For related structures and background, see: Addison *et al.* (2008 ▶); Anderson *et al.* (2006 ▶); Burgeson *et al.* (2004 ▶); Casper *et al.* (2002 ▶); Rodrigues *et al.* (2006 ▶); Szczepura *et al.* (2004 ▶); Trepanier *et al.* (1968 ▶). The synthesis of the title compound is described by Casper *et al.* (2004 ▶). For literature related to crystallographic analysis, see: Allen (2002 ▶); Bernstein *et al.* (1995 ▶); Boeyens (1978 ▶); Bruno *et al.* (2004 ▶); Cremer & Pople (1975 ▶); Etter *et al.* (1990 ▶); Macrae *et al.* (2008 ▶); Spek (2009 ▶).
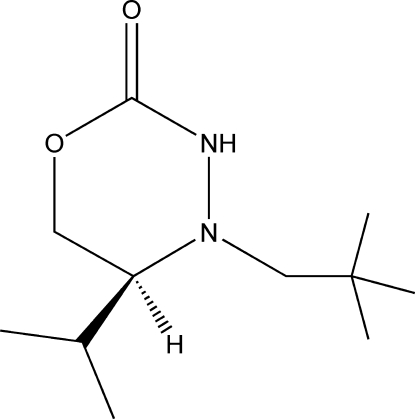


## Experimental

### 

#### Crystal data

C_11_H_22_N_2_O_2_
*M* *_r_* = 214.3
Monoclinic, 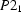

*a* = 17.0330 (18) Å
*b* = 11.2270 (12) Å
*c* = 17.404 (2) Å
β = 100.073 (2)°
*V* = 3276.9 (6) Å^3^
*Z* = 10
Mo *K*α radiation
μ = 0.08 mm^−1^
*T* = 193 K
0.6 × 0.32 × 0.27 mm

#### Data collection

Bruker P4/R4/SMART 1000 CCD diffractometer
Absorption correction: multi-scan (*SADABS* in *SAINT-Plus*; Bruker, 1999 ▶) *T*_min_ = 0.812, *T*_max_ = 0.943
16431 measured reflections
7042 independent reflections
4526 reflections with *I* > 2σ(*I*)
*R*_int_ = 0.044

#### Refinement

*R*[*F* ^2^ > 2σ(*F* ^2^)] = 0.052
*wR*(*F* ^2^) = 0.144
*S* = 1.03
7042 reflections
723 parameters
1 restraint
H atoms treated by a mixture of independent and constrained refinement
Δρ_max_ = 0.27 e Å^−3^
Δρ_min_ = −0.17 e Å^−3^

### 

Data collection: *SMART* (Bruker, 1999 ▶); cell refinement: *SAINT* (Bruker, 1999 ▶); data reduction: *SAINT*; program(s) used to solve structure: *SIR2004* (Burla *et al.*, 2005 ▶); program(s) used to refine structure: *SHELXL97* (Sheldrick, 2008 ▶); molecular graphics: *ORTEP-3 for Windows* (Farrugia, 1997 ▶); software used to prepare material for publication: *WinGX* (Farrugia, 1999 ▶), *publCIF* (Westrip, 2010) ▶ and *Mercury* (Macrae *et al.*, 2008 ▶).

## Supplementary Material

Crystal structure: contains datablocks global, I. DOI: 10.1107/S1600536810048798/zl2318sup1.cif

Structure factors: contains datablocks I. DOI: 10.1107/S1600536810048798/zl2318Isup2.hkl

Additional supplementary materials:  crystallographic information; 3D view; checkCIF report

**Fig. 4.**
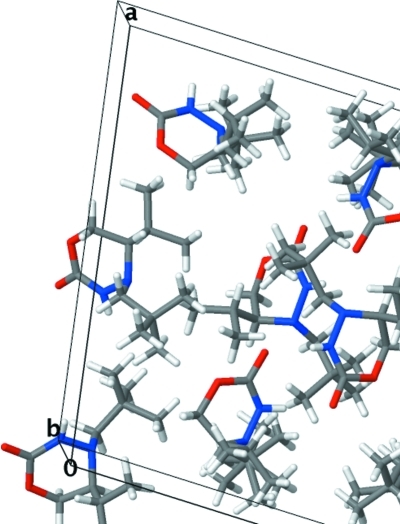
The Jmol enhanced figure of the title compound. The H-bonding situation can clearly be seen by manipulating the various radiobuttons.

Enhanced figure: interactive version of Fig. 4

## Figures and Tables

**Table 1:** Hydrogen-bond geometry (Å, °)

*D*—H⋯*A*	*D*—H	H⋯*A*	*D*⋯*A*	*D*—H⋯*A*
N23—H23⋯O55^i^	0.78 (4)	2.16 (4)	2.937 (5)	177 (5)
N43—H43⋯O35^ii^	0.88 (4)	1.99 (4)	2.868 (5)	175 (3)
N63—H63⋯O95^iii^	0.84 (4)	2.13 (4)	2.949 (5)	163 (3)
N83—H83⋯O75^iv^	0.89 (4)	2.04 (4)	2.926 (4)	175 (4)

Symmetry codes: (i) 
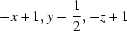
; (ii) 
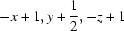
; (iii) 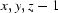; (iv) 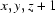.

**Fig. 1.**
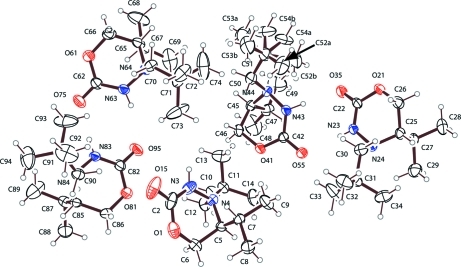
ORTEP view of the title compound showing the atomic numbering scheme of all five independent molecules in the asymmetric unit. Ellipsoids are shown at 50% probability displacement and hydrogen atoms are omitted for clarity.

**Fig. 2.**
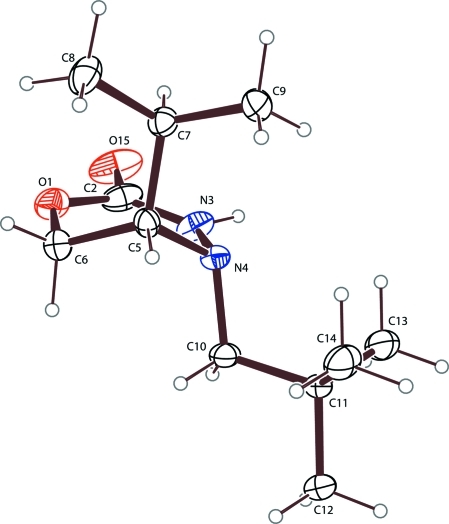
ORTEP view of the title compound showing the atomic numbering scheme for the best of the five independent molecules. Ellipsoids are shown at 50% probability displacement and hydrogen atoms are omitted for clarity.

**Fig. 3.**
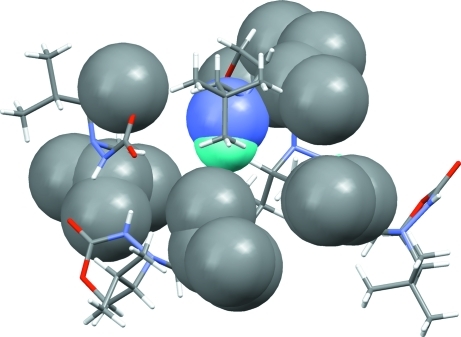
A view of the asymmetric unit in Mercury. The molecule that is not engaged in H-bonding has the N and H atoms shown in spacefill mode. Aliphatic substituents (C atoms shown in spacefill) surround this N–H and prohibit it from H-bonding with a carbonyl group.

**Fig. 5.**
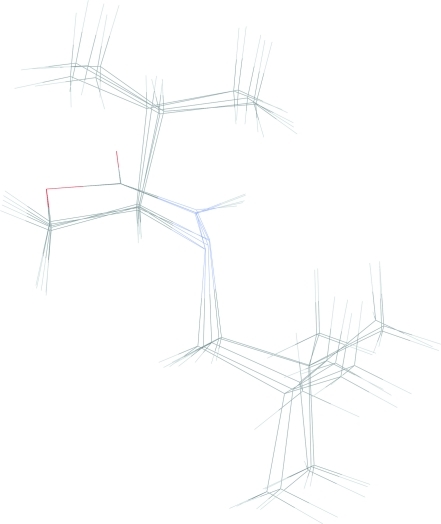
An overlay of the five molecules in the asymmetric unit shown in Mercury. There is no significant variation of the oxadiazinanone ring.

## supplementary crystallographic information

### Comment

The fundamental structure of 1,3,4-oxadiazinan-2-one compounds has been known for nearly forty years (Trepanier *et al.*, 1968). We are interested in studying the underlying factors for differing conformations of this heterocycle and for its use as a chiral auxiliary in aldol addition reactions (Casper *et al.*, 2002; Burgeson *et al.*, 2004).

The oxadiazinanone ring is well suited to accommodate H-bonded dimers of the *R*^2^_2_(8) type (Bernstein *et al.*, 1995; Etter *et al.*, 1990). The H-bond occurs most naturally between the carbonyl oxygen on the first molecule and the nitrogen containing the hydrogen atom on the second molecule, and *vice versa*. The result is an eight-membered ring in which two of the connections are H-bonds. These H-bonded dimers have a strong propensity to form in the oxadiazinanone system (Anderson *et al.*, 2006). A few examples of this type of H-bonding are seen in structures by Addison *et al.* (2008), Rodrigues *et al.* (2006), and Szczepura *et al.* (2004) (with REFCODES LOBVII, GEGZUO, and YAGJEW, respectively).

The title compound packs into a monoclinic, *P*2_1_, space group. This compound is unusual in that there are five molecules in the asymmetric unit (see Figure 1).  The best of these is illustrated in Figure 2. Of these five molecules, two pairs are engaged in *R*^2^_2_(8) type H-bonding. The fifth molecule does not engage in H-bonding because the donor atom is surrounded by neopentyl substituents, effectively blocking any H-bonds to an acceptor atom. Figure 3 shows all five molecules in a wireframe style, with key atoms shown in a space-fill style, to highlight how the N—H is hemmed in by neopentyl substituents. Another, interactive, view in Figure 4 shows how inaccessible the donor N really is.

The number of crystallographically independent molecules in the asymmetric unit also warrants notice. Most of the structures in the Cambridge Structural Database have a *Z*' less than or equal to 1. According to the August 2010 edition of the CSD (Allen, 2002), 91.1% of the structures have a *Z*' less than or equal to 1. As *Z*' increases, the percentage of structures decreases (*Z*'=2, 7.7%; *Z*'=3, 0.42%; *Z*'=4, 0.40%). Only 0.014% (N=73) of structures in the CSD have a *Z*'=5, which is a property of this structure.

Since the conformation of the heterocycle is of some interest, structure overlays were conducted in Mercury (Macrae *et al.*, 2008). Surprisingly, all five heterocycles exhibit the same conformation (seen in Figure 5); there is some swing observed in the isopropyl and neopentyl groups, but that deviation does not require the heterocycle to assume a different position in the crystal array. A Cremer-Pople ring analysis (Cremer & Pople, 1975; Boeyens, 1978), performed in *PLATON* (Spek, 2009), shows that all five molecules exhibit the ^5^E ring conformation, which is an envelope conformation with C5 as the flap apex. The data for the five oxadiazinanone rings are as follows: Ring 1 (O1—C6) - Q=0.511 (4); θ=56.7 (5)°; Φ=251.2 (5)°, Ring 2 (O21—C26) - Q=0.499 (4); θ=53.7 (5)°; Φ=253.9 (5)°, Ring 3 (O41—C46) - Q=0.510 (4); θ=56.7 (4)°; Φ=246.5 (5)°, Ring 4 (O61—C66) - Q=0.492 (5); θ=55.6 (5)°; Φ=239.9 (6)°, Ring 5 (O81—C86) - Q=0.500 (4); θ=55.9 (5)°; Φ=236.4 (5)°.

Upon conducting a *Mogul* geometry check (Bruno *et al.*, 2004), the N4—C10 (1.490 (5) Å) and N24—C30 bonds (1.488 (5) Å) were flagged as being outside the typical range for a C—N bond (mean 1.46 ±0.01 Å). This bond is the one between the N atom of the heterocycle and the neopentyl substituent. This bond in the other three crystallographically independent molecules is not flagged. The bonds C50—C51 (1.508 (7) Å) and C90—C91 (1.507 (6) Å) were also flagged as unusual compared to the mean of 1.54 ±0.01 Å. One last bond, N84—C85, was considered unusual with a value of 1.463 (5) Å *versus* a mean of 1.49 ±0.01 Å. There is no obvious particular chemical or crystallographic explanation for these deviations. However, they still appear to be within an acceptable range. All angles were reported to be within normal limits.

### Experimental

The title compound was prepared as previously reported (Casper *et al.* 2004). Single crystals were grown by vapor diffusion of hexane into an ethyl acetate solution of the title compound.

### Refinement

All non-H atoms were refined anisotropically. All H atoms were initially identified through difference Fourier syntheses then removed and included in the refinement in the riding-model approximation (C–H = 0.98, 0.99 and 1.00 Å for CH_3_, CH_2_, and CH; *U*_iso_(H) = 1.2U_eq_(C) except for methyl groups, where *U*_iso_(H) = 1.5U_eq_(C)).

### Crystal data

**Table:**

C_11_H_22_N_2_O_2_	*F*(000) = 1180
*M**_r_* = 214.3	*D*_x_ = 1.086 Mg m^−^^3^
Monoclinic, *P*2_1_	Mo *K*α radiation, λ = 0.71073 Å
Hall symbol: P 2yb	Cell parameters from 6729 reflections
*a* = 17.0330 (18) Å	θ = 2.4–22.8°
*b* = 11.2270 (12) Å	µ = 0.08 mm^−^^1^
*c* = 17.404 (2) Å	*T* = 193 K
β = 100.073 (2)°	Prism, colourless
*V* = 3276.9 (6) Å^3^	0.6 × 0.32 × 0.27 mm
*Z* = 10	

### Data collection

**Table:**

Bruker P4/R4/SMART 1000 CCD diffractometer	4526 reflections with *I* > 2σ(*I*)
ω scans	*R*_int_ = 0.044
Absorption correction: multi-scan (*SADABS* in *SAINT-Plus*; Bruker, 1999)	θ_max_ = 26.4°, θ_min_ = 1.2°
*T*_min_ = 0.812, *T*_max_ = 0.943	*h* = −21→21
16431 measured reflections	*k* = −11→14
7042 independent reflections	*l* = −19→21

### Refinement

**Table:**

Refinement on *F*^2^	1 restraint
Least-squares matrix: full	H atoms treated by a mixture of independent and constrained refinement
*R*[*F*^2^ > 2σ(*F*^2^)] = 0.052	*w* = 1/[σ^2^(*F*_o_^2^) + (0.0763*P*)^2^] where *P* = (*F*_o_^2^ + 2*F*_c_^2^)/3
*wR*(*F*^2^) = 0.144	(Δ/σ)_max_ < 0.001
*S* = 1.03	Δρ_max_ = 0.27 e Å^−^^3^
7042 reflections	Δρ_min_ = −0.17 e Å^−^^3^
723 parameters	

### Special details

**Table:**

Geometry. All e.s.d.'s (except the e.s.d. in the dihedral angle between two l.s. planes) are estimated using the full covariance matrix. The cell e.s.d.'s are taken into account individually in the estimation of e.s.d.'s in distances, angles and torsion angles; correlations between e.s.d.'s in cell parameters are only used when they are defined by crystal symmetry. An approximate (isotropic) treatment of cell e.s.d.'s is used for estimating e.s.d.'s involving l.s. planes.

### Fractional atomic coordinates and isotropic or equivalent isotropic displacement parameters (Å^2^)

**Table:**

	*x*	*y*	*z*	*U*_iso_*/*U*_eq_	Occ. (<1)
O1	0.4885 (2)	0.4683 (3)	−0.06758 (17)	0.0693 (9)	
C2	0.4247 (3)	0.4157 (5)	−0.0465 (3)	0.0679 (13)	
N3	0.4016 (2)	0.4554 (4)	0.0188 (3)	0.0625 (10)	
N4	0.44061 (17)	0.5387 (3)	0.07359 (18)	0.0427 (7)	
C5	0.5244 (2)	0.5438 (3)	0.0642 (2)	0.0418 (9)	
H5	0.5489	0.6146	0.0941	0.05*	
C6	0.5273 (3)	0.5655 (4)	−0.0204 (2)	0.0585 (11)	
H6A	0.5	0.6414	−0.0373	0.07*	
H6B	0.5834	0.572	−0.0278	0.07*	
C7	0.5696 (2)	0.4339 (4)	0.0995 (2)	0.0472 (9)	
H7	0.5419	0.3622	0.0737	0.057*	
C8	0.6560 (2)	0.4309 (5)	0.0861 (3)	0.0677 (13)	
H8A	0.6821	0.3586	0.1096	0.102*	
H8B	0.6845	0.5011	0.1102	0.102*	
H8C	0.6568	0.4311	0.0299	0.102*	
C9	0.5678 (3)	0.4252 (5)	0.1864 (3)	0.0690 (13)	
H9A	0.597	0.3539	0.2078	0.103*	
H9B	0.5124	0.4199	0.1945	0.103*	
H9C	0.5929	0.4961	0.213	0.103*	
C10	0.3986 (2)	0.6556 (3)	0.0666 (2)	0.0505 (10)	
H10A	0.3527	0.6517	0.023	0.061*	
H10B	0.4354	0.7179	0.0539	0.061*	
C11	0.3688 (2)	0.6901 (3)	0.1414 (2)	0.0443 (9)	
C12	0.3260 (3)	0.8094 (4)	0.1264 (3)	0.0634 (12)	
H12A	0.2807	0.8009	0.0834	0.095*	
H12B	0.3631	0.8691	0.1124	0.095*	
H12C	0.3067	0.835	0.1736	0.095*	
C13	0.3113 (3)	0.5967 (4)	0.1610 (3)	0.0792 (15)	
H13A	0.2668	0.5885	0.1174	0.119*	
H13B	0.291	0.6209	0.208	0.119*	
H13C	0.3391	0.5202	0.1705	0.119*	
C14	0.4384 (3)	0.7049 (5)	0.2076 (3)	0.0706 (13)	
H14A	0.4751	0.7651	0.1934	0.106*	
H14B	0.4665	0.6288	0.2175	0.106*	
H14C	0.4189	0.7304	0.2547	0.106*	
O15	0.3927 (2)	0.3348 (4)	−0.0854 (3)	0.1060 (15)	
H3	0.365 (3)	0.416 (5)	0.035 (3)	0.073 (16)*	
O21	0.60240 (16)	0.0228 (3)	0.35395 (17)	0.0629 (8)	
C22	0.6241 (2)	0.0932 (4)	0.4166 (2)	0.0528 (11)	
N23	0.56820 (19)	0.1295 (3)	0.4561 (2)	0.0483 (9)	
N24	0.48479 (16)	0.1070 (3)	0.43763 (17)	0.0439 (7)	
C25	0.4650 (2)	0.0769 (4)	0.3543 (2)	0.0477 (10)	
H25	0.4099	0.0429	0.3446	0.057*	
C26	0.5212 (2)	−0.0197 (4)	0.3368 (3)	0.0602 (11)	
H26A	0.5153	−0.091	0.3688	0.072*	
H26B	0.5077	−0.0425	0.2811	0.072*	
C27	0.4643 (2)	0.1884 (4)	0.3030 (2)	0.0557 (10)	
H27	0.519	0.2241	0.3128	0.067*	
C28	0.4435 (3)	0.1552 (6)	0.2171 (3)	0.0893 (17)	
H28A	0.4816	0.0958	0.2047	0.134*	
H28B	0.4461	0.2264	0.185	0.134*	
H28C	0.3895	0.122	0.2061	0.134*	
C29	0.4062 (3)	0.2798 (5)	0.3235 (3)	0.0782 (14)	
H29A	0.4067	0.3501	0.2902	0.117*	
H29B	0.4217	0.3029	0.3783	0.117*	
H29C	0.3524	0.2456	0.3152	0.117*	
C30	0.4607 (3)	0.0152 (4)	0.4906 (3)	0.0594 (11)	
H30A	0.5086	−0.0121	0.527	0.071*	
H30B	0.4383	−0.0543	0.4592	0.071*	
C31	0.3996 (2)	0.0603 (4)	0.5375 (2)	0.0539 (11)	
C32	0.3795 (3)	−0.0402 (6)	0.5887 (3)	0.0896 (17)	
H32A	0.4276	−0.0641	0.625	0.134*	
H32B	0.359	−0.1083	0.556	0.134*	
H32C	0.3388	−0.0133	0.6183	0.134*	
C33	0.4337 (4)	0.1663 (6)	0.5866 (3)	0.115 (3)	
H33A	0.4822	0.142	0.6221	0.172*	
H33B	0.3943	0.1952	0.6171	0.172*	
H33C	0.4465	0.2301	0.5524	0.172*	
C34	0.3249 (3)	0.0960 (5)	0.4830 (3)	0.0831 (16)	
H34A	0.3368	0.1618	0.4499	0.125*	
H34B	0.2846	0.1215	0.5134	0.125*	
H34C	0.3046	0.0279	0.4502	0.125*	
O35	0.69335 (16)	0.1255 (4)	0.43234 (17)	0.0761 (10)	
H23	0.584 (2)	0.169 (4)	0.492 (3)	0.051 (13)*	
O41	0.26808 (14)	0.8230 (3)	0.31233 (16)	0.0557 (7)	
C42	0.2958 (2)	0.7617 (4)	0.3774 (2)	0.0442 (9)	
N43	0.24610 (18)	0.6941 (3)	0.4097 (2)	0.0474 (8)	
N44	0.16241 (17)	0.6874 (3)	0.38521 (18)	0.0481 (8)	
C45	0.1355 (2)	0.7981 (4)	0.3434 (2)	0.0526 (11)	
H45	0.0787	0.7861	0.3181	0.063*	
C46	0.1835 (2)	0.8159 (5)	0.2790 (2)	0.0596 (11)	
H46A	0.1741	0.7486	0.2418	0.071*	
H46B	0.1664	0.8902	0.2502	0.071*	
C47	0.1371 (2)	0.9001 (4)	0.4006 (3)	0.0637 (12)	
H47	0.193	0.9072	0.4297	0.076*	
C48	0.1156 (5)	1.0160 (7)	0.3609 (5)	0.134 (3)	
H48A	0.1172	1.079	0.4001	0.201*	
H48B	0.0617	1.0109	0.3301	0.201*	
H48C	0.1536	1.0345	0.3264	0.201*	
C49	0.0835 (4)	0.8758 (7)	0.4601 (4)	0.117 (2)	
H49A	0.0982	0.7995	0.486	0.176*	
H49B	0.0278	0.8726	0.4335	0.176*	
H49C	0.0898	0.9397	0.499	0.176*	
C50	0.1397 (3)	0.5776 (5)	0.3403 (3)	0.0758 (14)	
H50A	0.1791	0.5632	0.3057	0.091*	
H50B	0.0873	0.5906	0.3066	0.091*	
C51	0.1346 (3)	0.4674 (5)	0.3887 (3)	0.0726 (14)	
C52A	0.2148 (10)	0.4158 (14)	0.4062 (10)	0.099 (5)	0.5
H52A	0.2136	0.3437	0.4376	0.148*	0.5
H52B	0.2515	0.4738	0.4352	0.148*	0.5
H52C	0.2328	0.3956	0.3573	0.148*	0.5
C53A	0.0741 (8)	0.3832 (14)	0.3425 (10)	0.099 (5)	0.5
H53A	0.0701	0.3109	0.3731	0.149*	0.5
H53B	0.0914	0.362	0.2934	0.149*	0.5
H53C	0.022	0.4224	0.3312	0.149*	0.5
C54A	0.1074 (11)	0.5004 (16)	0.4667 (10)	0.116 (6)	0.5
H54A	0.1044	0.4281	0.4975	0.175*	0.5
H54B	0.0547	0.5382	0.4556	0.175*	0.5
H54C	0.1458	0.5558	0.4961	0.175*	0.5
C52B	0.2081 (13)	0.4386 (14)	0.4517 (9)	0.110 (6)	0.5
H52D	0.2549	0.427	0.4268	0.164*	0.5
H52E	0.198	0.3657	0.4794	0.164*	0.5
H52F	0.218	0.5048	0.4889	0.164*	0.5
C53B	0.1269 (12)	0.3535 (13)	0.3335 (9)	0.110 (5)	0.5
H53D	0.1754	0.345	0.311	0.164*	0.5
H53E	0.0808	0.3631	0.2914	0.164*	0.5
H53F	0.1195	0.2823	0.364	0.164*	0.5
C54B	0.0622 (11)	0.4802 (16)	0.4233 (13)	0.130 (7)	0.5
H54D	0.0563	0.4103	0.4556	0.196*	0.5
H54E	0.0155	0.4868	0.3817	0.196*	0.5
H54F	0.0665	0.552	0.4558	0.196*	0.5
O55	0.36702 (14)	0.7706 (3)	0.40520 (15)	0.0533 (7)	
H43	0.262 (2)	0.673 (3)	0.459 (2)	0.041 (10)*	
O61	−0.06692 (15)	0.3012 (3)	−0.09221 (17)	0.0681 (9)	
C62	−0.0031 (2)	0.3696 (4)	−0.0965 (3)	0.0547 (11)	
N63	0.0563 (2)	0.3695 (3)	−0.03533 (19)	0.0466 (8)	
N64	0.05885 (16)	0.3060 (3)	0.03561 (18)	0.0431 (7)	
C65	−0.0238 (2)	0.2907 (4)	0.0483 (3)	0.0546 (11)	
H65	−0.0228	0.234	0.0928	0.066*	
C66	−0.0709 (2)	0.2334 (5)	−0.0230 (3)	0.0670 (14)	
H66A	−0.05	0.1522	−0.0289	0.08*	
H66B	−0.1273	0.2259	−0.0165	0.08*	
C67	−0.0578 (2)	0.4086 (4)	0.0716 (3)	0.0648 (13)	
H67	−0.0659	0.4619	0.0248	0.078*	
C68	−0.1395 (3)	0.3876 (6)	0.0954 (4)	0.118 (3)	
H68A	−0.1747	0.3467	0.053	0.177*	
H68B	−0.163	0.4643	0.1057	0.177*	
H68C	−0.1327	0.3383	0.1426	0.177*	
C69	−0.0022 (4)	0.4701 (7)	0.1355 (4)	0.112 (2)	
H69A	−0.0263	0.545	0.1487	0.168*	
H69B	0.0483	0.4869	0.118	0.168*	
H69C	0.0077	0.4187	0.1817	0.168*	
C70	0.1029 (2)	0.1934 (4)	0.0357 (2)	0.0482 (9)	
H70A	0.1085	0.1742	−0.0186	0.058*	
H70B	0.0713	0.129	0.0543	0.058*	
C71	0.1850 (2)	0.1955 (4)	0.0860 (3)	0.0574 (11)	
C72	0.2217 (3)	0.0725 (4)	0.0846 (4)	0.0850 (17)	
H72A	0.1869	0.0136	0.1031	0.128*	
H72B	0.274	0.0717	0.1186	0.128*	
H72C	0.228	0.0529	0.0311	0.128*	
C73	0.2369 (3)	0.2847 (6)	0.0590 (6)	0.135 (3)	
H73A	0.2433	0.2662	0.0054	0.203*	
H73B	0.2891	0.2838	0.0931	0.203*	
H73C	0.2129	0.3639	0.0604	0.203*	
C74	0.1731 (5)	0.2199 (8)	0.1695 (4)	0.151 (4)	
H74A	0.1381	0.1589	0.1855	0.227*	
H74B	0.1488	0.2985	0.1722	0.227*	
H74C	0.2248	0.2179	0.2045	0.227*	
O75	−0.00226 (17)	0.4282 (4)	−0.15440 (18)	0.0753 (10)	
H63	0.092 (2)	0.422 (4)	−0.030 (2)	0.039 (11)*	
O81	0.26791 (18)	0.5980 (3)	0.87572 (16)	0.0671 (8)	
C82	0.2004 (3)	0.5404 (4)	0.8848 (2)	0.0540 (10)	
N83	0.1615 (2)	0.4771 (3)	0.82481 (18)	0.0471 (8)	
N84	0.18487 (17)	0.4653 (3)	0.75098 (17)	0.0410 (7)	
C85	0.2253 (2)	0.5750 (3)	0.7346 (2)	0.0445 (9)	
H85	0.248	0.5615	0.6862	0.053*	
C86	0.2943 (3)	0.5952 (4)	0.8008 (3)	0.0614 (12)	
H86A	0.3339	0.5305	0.801	0.074*	
H86B	0.3207	0.6715	0.7926	0.074*	
C87	0.1666 (3)	0.6781 (4)	0.7187 (3)	0.0571 (11)	
H87	0.144	0.6927	0.7671	0.068*	
C88	0.2050 (4)	0.7922 (5)	0.6981 (3)	0.0865 (16)	
H88A	0.1646	0.8551	0.6882	0.13*	
H88B	0.2285	0.7798	0.6512	0.13*	
H88C	0.2468	0.8159	0.7415	0.13*	
C89	0.0988 (3)	0.6471 (5)	0.6541 (4)	0.111 (2)	
H89A	0.0741	0.5724	0.6668	0.167*	
H89B	0.1191	0.6376	0.6051	0.167*	
H89C	0.059	0.711	0.6484	0.167*	
C90	0.2337 (2)	0.3574 (4)	0.7488 (3)	0.0539 (10)	
H90A	0.2631	0.3415	0.8021	0.065*	
H90B	0.2736	0.3729	0.7149	0.065*	
C91	0.1870 (3)	0.2476 (4)	0.7195 (3)	0.0619 (12)	
C92	0.2443 (4)	0.1445 (5)	0.7213 (5)	0.114 (2)	
H92A	0.2651	0.1229	0.7757	0.172*	
H92B	0.2886	0.1676	0.6953	0.172*	
H92C	0.2164	0.076	0.6942	0.172*	
C93	0.1213 (5)	0.2203 (5)	0.7643 (5)	0.126 (3)	
H93A	0.1442	0.2063	0.8192	0.189*	
H93B	0.0925	0.1489	0.7425	0.189*	
H93C	0.0843	0.2878	0.7603	0.189*	
C94	0.1492 (5)	0.2668 (7)	0.6349 (4)	0.129 (3)	
H94A	0.1189	0.1956	0.615	0.193*	
H94B	0.1909	0.2818	0.6038	0.193*	
H94C	0.1131	0.3355	0.631	0.193*	
O95	0.1774 (2)	0.5505 (3)	0.94701 (17)	0.0708 (9)	
H83	0.111 (3)	0.459 (4)	0.828 (2)	0.057 (12)*	

### Atomic displacement parameters (Å^2^)

**Table:**

	*U*^11^	*U*^22^	*U*^33^	*U*^12^	*U*^13^	*U*^23^
O1	0.094 (2)	0.066 (2)	0.0474 (19)	0.018 (2)	0.0094 (16)	−0.0103 (16)
C2	0.062 (3)	0.061 (3)	0.071 (3)	0.019 (3)	−0.016 (3)	−0.021 (3)
N3	0.0434 (19)	0.051 (2)	0.091 (3)	0.0002 (18)	0.008 (2)	−0.019 (2)
N4	0.0435 (17)	0.0341 (17)	0.0500 (19)	0.0035 (14)	0.0072 (14)	−0.0024 (14)
C5	0.046 (2)	0.039 (2)	0.042 (2)	−0.0077 (17)	0.0120 (16)	−0.0003 (16)
C6	0.072 (3)	0.057 (3)	0.051 (3)	0.009 (2)	0.023 (2)	0.007 (2)
C7	0.041 (2)	0.052 (3)	0.048 (2)	0.0035 (18)	0.0075 (17)	0.0005 (18)
C8	0.044 (2)	0.096 (4)	0.065 (3)	0.011 (2)	0.016 (2)	−0.002 (3)
C9	0.073 (3)	0.083 (3)	0.056 (3)	0.028 (3)	0.024 (2)	0.024 (2)
C10	0.061 (2)	0.043 (2)	0.045 (2)	0.0172 (19)	0.0050 (19)	0.0033 (17)
C11	0.055 (2)	0.035 (2)	0.045 (2)	0.0106 (18)	0.0135 (17)	0.0044 (17)
C12	0.085 (3)	0.047 (2)	0.063 (3)	0.024 (2)	0.025 (2)	0.005 (2)
C13	0.085 (3)	0.051 (3)	0.115 (4)	0.007 (3)	0.054 (3)	0.008 (3)
C14	0.090 (3)	0.073 (3)	0.047 (3)	0.021 (3)	0.004 (2)	−0.013 (2)
O15	0.095 (3)	0.083 (3)	0.121 (3)	0.017 (2)	−0.034 (2)	−0.060 (2)
O21	0.0514 (16)	0.087 (2)	0.0508 (18)	0.0006 (16)	0.0119 (13)	−0.0258 (16)
C22	0.045 (2)	0.077 (3)	0.037 (2)	0.001 (2)	0.0083 (17)	−0.013 (2)
N23	0.0409 (18)	0.066 (2)	0.037 (2)	−0.0028 (17)	0.0055 (15)	−0.0118 (17)
N24	0.0366 (16)	0.054 (2)	0.0412 (18)	−0.0047 (14)	0.0076 (13)	−0.0021 (15)
C25	0.039 (2)	0.056 (3)	0.046 (2)	−0.0114 (18)	0.0031 (16)	−0.0085 (18)
C26	0.057 (2)	0.067 (3)	0.057 (3)	−0.011 (2)	0.011 (2)	−0.018 (2)
C27	0.050 (2)	0.074 (3)	0.040 (2)	−0.004 (2)	0.0008 (17)	0.000 (2)
C28	0.094 (4)	0.119 (5)	0.048 (3)	0.002 (4)	−0.007 (3)	−0.007 (3)
C29	0.081 (3)	0.081 (4)	0.072 (3)	0.015 (3)	0.012 (3)	0.017 (3)
C30	0.060 (2)	0.057 (3)	0.065 (3)	0.006 (2)	0.020 (2)	0.011 (2)
C31	0.060 (3)	0.054 (3)	0.052 (2)	−0.013 (2)	0.021 (2)	−0.0082 (19)
C32	0.108 (4)	0.097 (4)	0.075 (4)	−0.007 (4)	0.049 (3)	0.012 (3)
C33	0.141 (5)	0.138 (6)	0.080 (4)	−0.077 (5)	0.061 (4)	−0.058 (4)
C34	0.056 (3)	0.086 (4)	0.115 (4)	0.002 (3)	0.036 (3)	0.010 (3)
O35	0.0401 (16)	0.135 (3)	0.0543 (19)	−0.0083 (18)	0.0124 (13)	−0.0325 (19)
O41	0.0426 (14)	0.078 (2)	0.0464 (16)	0.0020 (14)	0.0070 (12)	0.0207 (14)
C42	0.038 (2)	0.053 (2)	0.042 (2)	0.0055 (19)	0.0077 (17)	0.0018 (18)
N43	0.0388 (17)	0.061 (2)	0.041 (2)	0.0029 (16)	0.0049 (14)	0.0135 (17)
N44	0.0369 (16)	0.057 (2)	0.047 (2)	−0.0061 (16)	−0.0007 (14)	0.0031 (16)
C45	0.035 (2)	0.064 (3)	0.056 (3)	0.0010 (19)	0.0001 (17)	0.008 (2)
C46	0.049 (2)	0.075 (3)	0.050 (3)	0.004 (2)	−0.0031 (19)	0.018 (2)
C47	0.043 (2)	0.062 (3)	0.086 (4)	0.009 (2)	0.009 (2)	0.009 (3)
C48	0.167 (7)	0.094 (5)	0.138 (7)	0.065 (5)	0.018 (5)	0.020 (5)
C49	0.125 (5)	0.121 (6)	0.124 (6)	0.006 (4)	0.071 (5)	−0.018 (4)
C50	0.088 (4)	0.079 (4)	0.055 (3)	−0.029 (3)	−0.003 (2)	−0.001 (3)
C51	0.089 (4)	0.065 (3)	0.059 (3)	−0.018 (3)	0.003 (3)	−0.002 (3)
C52A	0.108 (10)	0.055 (7)	0.118 (14)	0.012 (7)	−0.021 (11)	−0.014 (9)
C53A	0.088 (9)	0.077 (11)	0.121 (12)	−0.021 (8)	−0.014 (9)	0.000 (8)
C54A	0.135 (15)	0.111 (12)	0.113 (13)	−0.048 (11)	0.048 (10)	0.036 (10)
C52B	0.179 (16)	0.056 (9)	0.082 (11)	−0.013 (9)	−0.010 (12)	0.012 (7)
C53B	0.176 (17)	0.058 (8)	0.084 (9)	−0.016 (11)	−0.005 (12)	−0.015 (6)
C54B	0.124 (14)	0.102 (11)	0.18 (2)	−0.054 (11)	0.081 (13)	−0.015 (13)
O55	0.0359 (14)	0.0705 (19)	0.0539 (17)	0.0041 (13)	0.0090 (12)	0.0103 (14)
O61	0.0407 (15)	0.103 (3)	0.0592 (19)	−0.0076 (16)	0.0054 (13)	0.0175 (18)
C62	0.035 (2)	0.079 (3)	0.052 (3)	0.012 (2)	0.0137 (19)	0.015 (2)
N63	0.0367 (17)	0.058 (2)	0.047 (2)	0.0010 (18)	0.0126 (15)	0.0105 (16)
N64	0.0358 (16)	0.0461 (19)	0.0489 (19)	0.0097 (14)	0.0116 (13)	0.0123 (14)
C65	0.040 (2)	0.067 (3)	0.060 (3)	0.012 (2)	0.0177 (19)	0.029 (2)
C66	0.037 (2)	0.083 (4)	0.081 (3)	−0.004 (2)	0.012 (2)	0.022 (3)
C67	0.056 (2)	0.083 (3)	0.063 (3)	0.034 (2)	0.031 (2)	0.032 (3)
C68	0.088 (4)	0.130 (6)	0.159 (6)	0.046 (4)	0.083 (4)	0.063 (5)
C69	0.128 (5)	0.125 (5)	0.082 (4)	0.071 (5)	0.016 (4)	−0.016 (4)
C70	0.0370 (19)	0.040 (2)	0.067 (3)	0.0046 (18)	0.0091 (18)	−0.0033 (19)
C71	0.043 (2)	0.047 (2)	0.076 (3)	0.014 (2)	−0.0071 (19)	−0.004 (2)
C72	0.052 (3)	0.056 (3)	0.138 (5)	0.021 (2)	−0.011 (3)	−0.008 (3)
C73	0.045 (3)	0.078 (4)	0.267 (10)	−0.013 (3)	−0.019 (4)	0.047 (5)
C74	0.173 (7)	0.174 (8)	0.083 (5)	0.106 (7)	−0.041 (5)	−0.021 (5)
O75	0.0564 (17)	0.121 (3)	0.0505 (19)	0.0068 (18)	0.0150 (14)	0.0320 (19)
O81	0.081 (2)	0.073 (2)	0.0465 (17)	−0.0336 (17)	0.0100 (15)	−0.0064 (15)
C82	0.075 (3)	0.046 (2)	0.041 (2)	−0.014 (2)	0.013 (2)	0.0003 (19)
N83	0.053 (2)	0.050 (2)	0.0419 (19)	−0.0141 (17)	0.0191 (15)	−0.0075 (15)
N84	0.0451 (16)	0.0406 (17)	0.0418 (18)	−0.0061 (15)	0.0201 (13)	−0.0026 (14)
C85	0.050 (2)	0.044 (2)	0.044 (2)	−0.0114 (18)	0.0204 (18)	−0.0033 (17)
C86	0.059 (3)	0.067 (3)	0.060 (3)	−0.026 (2)	0.015 (2)	0.001 (2)
C87	0.081 (3)	0.043 (2)	0.054 (3)	−0.001 (2)	0.030 (2)	0.0006 (19)
C88	0.118 (4)	0.050 (3)	0.086 (4)	−0.022 (3)	0.004 (3)	0.008 (3)
C89	0.077 (4)	0.062 (4)	0.177 (7)	0.008 (3)	−0.027 (4)	0.022 (4)
C90	0.050 (2)	0.056 (3)	0.058 (3)	0.003 (2)	0.0167 (19)	0.004 (2)
C91	0.080 (3)	0.048 (3)	0.064 (3)	0.001 (2)	0.029 (2)	0.001 (2)
C92	0.151 (6)	0.057 (4)	0.150 (6)	0.030 (4)	0.067 (5)	0.006 (4)
C93	0.162 (6)	0.064 (4)	0.178 (7)	−0.050 (4)	0.102 (6)	−0.033 (4)
C94	0.201 (8)	0.088 (5)	0.082 (5)	−0.001 (5)	−0.017 (5)	−0.028 (4)
O95	0.107 (2)	0.068 (2)	0.0438 (18)	−0.0209 (19)	0.0316 (16)	−0.0081 (15)

### Geometric parameters (Å, °)

**Table:**

O1—C2	1.342 (6)	C51—C52A	1.465 (16)
O1—C6	1.453 (6)	C51—C54B	1.472 (17)
C2—O15	1.205 (5)	C51—C53A	1.521 (15)
C2—N3	1.342 (6)	C51—C52B	1.548 (18)
N3—N4	1.415 (5)	C51—C54A	1.554 (18)
N3—H3	0.85 (5)	C51—C53B	1.591 (15)
N4—C5	1.466 (4)	C52A—H52A	0.98
N4—C10	1.489 (5)	C52A—H52B	0.98
C5—C6	1.502 (5)	C52A—H52C	0.98
C5—C7	1.525 (5)	C53A—H53A	0.98
C5—H5	1	C53A—H53B	0.98
C6—H6A	0.99	C53A—H53C	0.98
C6—H6B	0.99	C54A—H54A	0.98
C7—C9	1.522 (6)	C54A—H54B	0.98
C7—C8	1.530 (5)	C54A—H54C	0.98
C7—H7	1	C52B—H52D	0.98
C8—H8A	0.98	C52B—H52E	0.98
C8—H8B	0.98	C52B—H52F	0.98
C8—H8C	0.98	C53B—H53D	0.98
C9—H9A	0.98	C53B—H53E	0.98
C9—H9B	0.98	C53B—H53F	0.98
C9—H9C	0.98	C54B—H54D	0.98
C10—C11	1.529 (5)	C54B—H54E	0.98
C10—H10A	0.99	C54B—H54F	0.98
C10—H10B	0.99	O61—C62	1.343 (5)
C11—C14	1.511 (6)	O61—C66	1.436 (5)
C11—C13	1.513 (6)	C62—O75	1.206 (5)
C11—C12	1.526 (5)	C62—N63	1.335 (5)
C12—H12A	0.98	N63—N64	1.420 (4)
C12—H12B	0.98	N63—H63	0.84 (4)
C12—H12C	0.98	N64—C70	1.470 (5)
C13—H13A	0.98	N64—C65	1.473 (4)
C13—H13B	0.98	C65—C66	1.501 (7)
C13—H13C	0.98	C65—C67	1.527 (6)
C14—H14A	0.98	C65—H65	1
C14—H14B	0.98	C66—H66A	0.99
C14—H14C	0.98	C66—H66B	0.99
O21—C22	1.345 (5)	C67—C69	1.498 (8)
O21—C26	1.444 (5)	C67—C68	1.538 (6)
C22—O35	1.219 (5)	C67—H67	1
C22—N23	1.331 (5)	C68—H68A	0.98
N23—N24	1.423 (4)	C68—H68B	0.98
N23—H23	0.78 (4)	C68—H68C	0.98
N24—C25	1.469 (5)	C69—H69A	0.98
N24—C30	1.487 (5)	C69—H69B	0.98
C25—C26	1.511 (6)	C69—H69C	0.98
C25—C27	1.537 (6)	C70—C71	1.514 (5)
C25—H25	1	C70—H70A	0.99
C26—H26A	0.99	C70—H70B	0.99
C26—H26B	0.99	C71—C73	1.467 (8)
C27—C29	1.512 (7)	C71—C72	1.518 (6)
C27—C28	1.521 (6)	C71—C74	1.527 (8)
C27—H27	1	C72—H72A	0.98
C28—H28A	0.98	C72—H72B	0.98
C28—H28B	0.98	C72—H72C	0.98
C28—H28C	0.98	C73—H73A	0.98
C29—H29A	0.98	C73—H73B	0.98
C29—H29B	0.98	C73—H73C	0.98
C29—H29C	0.98	C74—H74A	0.98
C30—C31	1.517 (5)	C74—H74B	0.98
C30—H30A	0.99	C74—H74C	0.98
C30—H30B	0.99	O81—C82	1.352 (5)
C31—C34	1.502 (7)	O81—C86	1.452 (5)
C31—C32	1.513 (7)	C82—O95	1.220 (5)
C31—C33	1.521 (7)	C82—N83	1.339 (5)
C32—H32A	0.98	N83—N84	1.417 (4)
C32—H32B	0.98	N83—H83	0.89 (4)
C32—H32C	0.98	N84—C85	1.463 (5)
C33—H33A	0.98	N84—C90	1.473 (5)
C33—H33B	0.98	C85—C86	1.513 (6)
C33—H33C	0.98	C85—C87	1.522 (6)
C34—H34A	0.98	C85—H85	1
C34—H34B	0.98	C86—H86A	0.99
C34—H34C	0.98	C86—H86B	0.99
O41—C42	1.339 (4)	C87—C89	1.504 (7)
O41—C46	1.459 (5)	C87—C88	1.510 (7)
C42—O55	1.229 (4)	C87—H87	1
C42—N43	1.332 (5)	C88—H88A	0.98
N43—N44	1.417 (4)	C88—H88B	0.98
N43—H43	0.88 (4)	C88—H88C	0.98
N44—C45	1.473 (5)	C89—H89A	0.98
N44—C50	1.473 (6)	C89—H89B	0.98
C45—C46	1.512 (6)	C89—H89C	0.98
C45—C47	1.513 (7)	C90—C91	1.507 (6)
C45—H45	1	C90—H90A	0.99
C46—H46A	0.99	C90—H90B	0.99
C46—H46B	0.99	C91—C93	1.503 (7)
C47—C48	1.489 (8)	C91—C92	1.511 (7)
C47—C49	1.520 (8)	C91—C94	1.516 (8)
C47—H47	1	C92—H92A	0.98
C48—H48A	0.98	C92—H92B	0.98
C48—H48B	0.98	C92—H92C	0.98
C48—H48C	0.98	C93—H93A	0.98
C49—H49A	0.98	C93—H93B	0.98
C49—H49B	0.98	C93—H93C	0.98
C49—H49C	0.98	C94—H94A	0.98
C50—C51	1.507 (7)	C94—H94B	0.98
C50—H50A	0.99	C94—H94C	0.98
C50—H50B	0.99		
			
C2—O1—C6	119.0 (3)	C54B—C51—C53A	74.9 (10)
O15—C2—N3	123.8 (6)	C50—C51—C53A	108.4 (7)
O15—C2—O1	119.1 (5)	C54B—C51—C52B	111.2 (12)
N3—C2—O1	117.1 (4)	C50—C51—C52B	116.6 (7)
C2—N3—N4	128.2 (4)	C53A—C51—C52B	129.3 (9)
C2—N3—H3	117 (3)	C52A—C51—C54A	108.8 (10)
N4—N3—H3	113 (4)	C50—C51—C54A	110.3 (7)
N3—N4—C5	107.6 (3)	C53A—C51—C54A	109.4 (10)
N3—N4—C10	111.4 (3)	C52B—C51—C54A	76.0 (10)
C5—N4—C10	114.8 (3)	C52A—C51—C53B	77.5 (10)
N4—C5—C6	108.3 (3)	C54B—C51—C53B	109.8 (11)
N4—C5—C7	110.9 (3)	C50—C51—C53B	109.2 (7)
C6—C5—C7	115.2 (3)	C52B—C51—C53B	103.4 (10)
N4—C5—H5	107.4	C54A—C51—C53B	135.5 (9)
C6—C5—H5	107.4	C51—C52A—H52A	109.5
C7—C5—H5	107.4	C51—C52A—H52B	109.5
O1—C6—C5	110.1 (3)	H52A—C52A—H52B	109.5
O1—C6—H6A	109.6	C51—C52A—H52C	109.5
C5—C6—H6A	109.6	H52A—C52A—H52C	109.5
O1—C6—H6B	109.6	H52B—C52A—H52C	109.5
C5—C6—H6B	109.6	C51—C53A—H53A	109.5
H6A—C6—H6B	108.2	C51—C53A—H53B	109.5
C9—C7—C5	110.8 (3)	H53A—C53A—H53B	109.5
C9—C7—C8	109.7 (3)	C51—C53A—H53C	109.5
C5—C7—C8	112.8 (3)	H53A—C53A—H53C	109.5
C9—C7—H7	107.8	H53B—C53A—H53C	109.5
C5—C7—H7	107.8	C51—C54A—H54A	109.5
C8—C7—H7	107.8	C51—C54A—H54B	109.5
C7—C8—H8A	109.5	H54A—C54A—H54B	109.5
C7—C8—H8B	109.5	C51—C54A—H54C	109.5
H8A—C8—H8B	109.5	H54A—C54A—H54C	109.5
C7—C8—H8C	109.5	H54B—C54A—H54C	109.5
H8A—C8—H8C	109.5	C51—C52B—H52D	109.5
H8B—C8—H8C	109.5	C51—C52B—H52E	109.5
C7—C9—H9A	109.5	H52D—C52B—H52E	109.5
C7—C9—H9B	109.5	C51—C52B—H52F	109.5
H9A—C9—H9B	109.5	H52D—C52B—H52F	109.5
C7—C9—H9C	109.5	H52E—C52B—H52F	109.5
H9A—C9—H9C	109.5	C51—C53B—H53D	109.5
H9B—C9—H9C	109.5	C51—C53B—H53E	109.5
N4—C10—C11	112.3 (3)	H53D—C53B—H53E	109.5
N4—C10—H10A	109.1	C51—C53B—H53F	109.5
C11—C10—H10A	109.1	H53D—C53B—H53F	109.5
N4—C10—H10B	109.1	H53E—C53B—H53F	109.5
C11—C10—H10B	109.1	C51—C54B—H54D	109.5
H10A—C10—H10B	107.9	C51—C54B—H54E	109.5
C14—C11—C13	110.9 (4)	H54D—C54B—H54E	109.5
C14—C11—C12	108.8 (4)	C51—C54B—H54F	109.5
C13—C11—C12	109.8 (3)	H54D—C54B—H54F	109.5
C14—C11—C10	110.1 (3)	H54E—C54B—H54F	109.5
C13—C11—C10	109.9 (4)	C62—O61—C66	120.5 (3)
C12—C11—C10	107.2 (3)	O75—C62—N63	123.2 (4)
C11—C12—H12A	109.5	O75—C62—O61	119.1 (4)
C11—C12—H12B	109.5	N63—C62—O61	117.7 (4)
H12A—C12—H12B	109.5	C62—N63—N64	126.6 (3)
C11—C12—H12C	109.5	C62—N63—H63	122 (3)
H12A—C12—H12C	109.5	N64—N63—H63	110 (3)
H12B—C12—H12C	109.5	N63—N64—C70	111.7 (3)
C11—C13—H13A	109.5	N63—N64—C65	107.8 (3)
C11—C13—H13B	109.5	C70—N64—C65	113.6 (3)
H13A—C13—H13B	109.5	N64—C65—C66	108.2 (3)
C11—C13—H13C	109.5	N64—C65—C67	110.8 (4)
H13A—C13—H13C	109.5	C66—C65—C67	114.8 (4)
H13B—C13—H13C	109.5	N64—C65—H65	107.6
C11—C14—H14A	109.5	C66—C65—H65	107.6
C11—C14—H14B	109.5	C67—C65—H65	107.6
H14A—C14—H14B	109.5	O61—C66—C65	111.7 (4)
C11—C14—H14C	109.5	O61—C66—H66A	109.3
H14A—C14—H14C	109.5	C65—C66—H66A	109.3
H14B—C14—H14C	109.5	O61—C66—H66B	109.3
C22—O21—C26	118.6 (3)	C65—C66—H66B	109.3
O35—C22—N23	123.0 (4)	H66A—C66—H66B	107.9
O35—C22—O21	118.3 (3)	C69—C67—C65	112.2 (4)
N23—C22—O21	118.6 (4)	C69—C67—C68	110.8 (5)
C22—N23—N24	127.2 (4)	C65—C67—C68	109.9 (5)
C22—N23—H23	114 (3)	C69—C67—H67	107.9
N24—N23—H23	118 (3)	C65—C67—H67	107.9
N23—N24—C25	108.1 (3)	C68—C67—H67	107.9
N23—N24—C30	110.9 (3)	C67—C68—H68A	109.5
C25—N24—C30	114.3 (3)	C67—C68—H68B	109.5
N24—C25—C26	108.7 (3)	H68A—C68—H68B	109.5
N24—C25—C27	111.3 (3)	C67—C68—H68C	109.5
C26—C25—C27	114.0 (3)	H68A—C68—H68C	109.5
N24—C25—H25	107.5	H68B—C68—H68C	109.5
C26—C25—H25	107.5	C67—C69—H69A	109.5
C27—C25—H25	107.5	C67—C69—H69B	109.5
O21—C26—C25	109.8 (3)	H69A—C69—H69B	109.5
O21—C26—H26A	109.7	C67—C69—H69C	109.5
C25—C26—H26A	109.7	H69A—C69—H69C	109.5
O21—C26—H26B	109.7	H69B—C69—H69C	109.5
C25—C26—H26B	109.7	N64—C70—C71	114.0 (3)
H26A—C26—H26B	108.2	N64—C70—H70A	108.8
C29—C27—C28	110.4 (4)	C71—C70—H70A	108.8
C29—C27—C25	110.7 (3)	N64—C70—H70B	108.8
C28—C27—C25	110.4 (4)	C71—C70—H70B	108.8
C29—C27—H27	108.4	H70A—C70—H70B	107.7
C28—C27—H27	108.4	C73—C71—C70	111.9 (4)
C25—C27—H27	108.4	C73—C71—C72	110.0 (4)
C27—C28—H28A	109.5	C70—C71—C72	108.5 (4)
C27—C28—H28B	109.5	C73—C71—C74	111.7 (6)
H28A—C28—H28B	109.5	C70—C71—C74	106.9 (4)
C27—C28—H28C	109.5	C72—C71—C74	107.5 (5)
H28A—C28—H28C	109.5	C71—C72—H72A	109.5
H28B—C28—H28C	109.5	C71—C72—H72B	109.5
C27—C29—H29A	109.5	H72A—C72—H72B	109.5
C27—C29—H29B	109.5	C71—C72—H72C	109.5
H29A—C29—H29B	109.5	H72A—C72—H72C	109.5
C27—C29—H29C	109.5	H72B—C72—H72C	109.5
H29A—C29—H29C	109.5	C71—C73—H73A	109.5
H29B—C29—H29C	109.5	C71—C73—H73B	109.5
N24—C30—C31	113.1 (3)	H73A—C73—H73B	109.5
N24—C30—H30A	109	C71—C73—H73C	109.5
C31—C30—H30A	109	H73A—C73—H73C	109.5
N24—C30—H30B	109	H73B—C73—H73C	109.5
C31—C30—H30B	109	C71—C74—H74A	109.5
H30A—C30—H30B	107.8	C71—C74—H74B	109.5
C34—C31—C32	108.5 (4)	H74A—C74—H74B	109.5
C34—C31—C30	109.5 (4)	C71—C74—H74C	109.5
C32—C31—C30	108.4 (4)	H74A—C74—H74C	109.5
C34—C31—C33	110.1 (5)	H74B—C74—H74C	109.5
C32—C31—C33	110.9 (4)	C82—O81—C86	119.8 (3)
C30—C31—C33	109.5 (4)	O95—C82—N83	123.3 (4)
C31—C32—H32A	109.5	O95—C82—O81	118.1 (4)
C31—C32—H32B	109.5	N83—C82—O81	118.6 (4)
H32A—C32—H32B	109.5	C82—N83—N84	125.2 (3)
C31—C32—H32C	109.5	C82—N83—H83	115 (3)
H32A—C32—H32C	109.5	N84—N83—H83	118 (3)
H32B—C32—H32C	109.5	N83—N84—C85	108.3 (3)
C31—C33—H33A	109.5	N83—N84—C90	110.5 (3)
C31—C33—H33B	109.5	C85—N84—C90	113.6 (3)
H33A—C33—H33B	109.5	N84—C85—C86	107.9 (3)
C31—C33—H33C	109.5	N84—C85—C87	111.1 (3)
H33A—C33—H33C	109.5	C86—C85—C87	115.0 (4)
H33B—C33—H33C	109.5	N84—C85—H85	107.5
C31—C34—H34A	109.5	C86—C85—H85	107.5
C31—C34—H34B	109.5	C87—C85—H85	107.5
H34A—C34—H34B	109.5	O81—C86—C85	111.4 (3)
C31—C34—H34C	109.5	O81—C86—H86A	109.3
H34A—C34—H34C	109.5	C85—C86—H86A	109.3
H34B—C34—H34C	109.5	O81—C86—H86B	109.3
C42—O41—C46	119.1 (3)	C85—C86—H86B	109.3
O55—C42—N43	122.5 (4)	H86A—C86—H86B	108
O55—C42—O41	118.0 (3)	C89—C87—C88	109.1 (4)
N43—C42—O41	119.6 (3)	C89—C87—C85	110.7 (4)
C42—N43—N44	125.8 (3)	C88—C87—C85	113.0 (4)
C42—N43—H43	117 (2)	C89—C87—H87	108
N44—N43—H43	113 (2)	C88—C87—H87	108
N43—N44—C45	108.2 (3)	C85—C87—H87	108
N43—N44—C50	111.2 (4)	C87—C88—H88A	109.5
C45—N44—C50	114.5 (3)	C87—C88—H88B	109.5
N44—C45—C46	108.4 (3)	H88A—C88—H88B	109.5
N44—C45—C47	110.2 (3)	C87—C88—H88C	109.5
C46—C45—C47	116.0 (4)	H88A—C88—H88C	109.5
N44—C45—H45	107.3	H88B—C88—H88C	109.5
C46—C45—H45	107.3	C87—C89—H89A	109.5
C47—C45—H45	107.3	C87—C89—H89B	109.5
O41—C46—C45	109.6 (3)	H89A—C89—H89B	109.5
O41—C46—H46A	109.7	C87—C89—H89C	109.5
C45—C46—H46A	109.7	H89A—C89—H89C	109.5
O41—C46—H46B	109.7	H89B—C89—H89C	109.5
C45—C46—H46B	109.7	N84—C90—C91	114.5 (3)
H46A—C46—H46B	108.2	N84—C90—H90A	108.6
C48—C47—C45	112.4 (5)	C91—C90—H90A	108.6
C48—C47—C49	110.1 (5)	N84—C90—H90B	108.6
C45—C47—C49	111.6 (4)	C91—C90—H90B	108.6
C48—C47—H47	107.5	H90A—C90—H90B	107.6
C45—C47—H47	107.5	C93—C91—C90	112.8 (4)
C49—C47—H47	107.5	C93—C91—C92	111.7 (5)
C47—C48—H48A	109.5	C90—C91—C92	108.6 (4)
C47—C48—H48B	109.5	C93—C91—C94	107.7 (6)
H48A—C48—H48B	109.5	C90—C91—C94	108.8 (4)
C47—C48—H48C	109.5	C92—C91—C94	107.0 (5)
H48A—C48—H48C	109.5	C91—C92—H92A	109.5
H48B—C48—H48C	109.5	C91—C92—H92B	109.5
C47—C49—H49A	109.5	H92A—C92—H92B	109.5
C47—C49—H49B	109.5	C91—C92—H92C	109.5
H49A—C49—H49B	109.5	H92A—C92—H92C	109.5
C47—C49—H49C	109.5	H92B—C92—H92C	109.5
H49A—C49—H49C	109.5	C91—C93—H93A	109.5
H49B—C49—H49C	109.5	C91—C93—H93B	109.5
N44—C50—C51	115.2 (4)	H93A—C93—H93B	109.5
N44—C50—H50A	108.5	C91—C93—H93C	109.5
C51—C50—H50A	108.5	H93A—C93—H93C	109.5
N44—C50—H50B	108.5	H93B—C93—H93C	109.5
C51—C50—H50B	108.5	C91—C94—H94A	109.5
H50A—C50—H50B	107.5	C91—C94—H94B	109.5
C52A—C51—C54B	140.4 (13)	H94A—C94—H94B	109.5
C52A—C51—C50	107.3 (8)	C91—C94—H94C	109.5
C54B—C51—C50	106.5 (9)	H94A—C94—H94C	109.5
C52A—C51—C53A	112.6 (9)	H94B—C94—H94C	109.5
			
C6—O1—C2—O15	−179.7 (4)	N44—C45—C47—C48	176.2 (5)
C6—O1—C2—N3	−1.4 (6)	C46—C45—C47—C48	52.6 (6)
O15—C2—N3—N4	171.3 (4)	N44—C45—C47—C49	−59.5 (5)
O1—C2—N3—N4	−6.9 (7)	C46—C45—C47—C49	176.8 (4)
C2—N3—N4—C5	−20.3 (6)	N43—N44—C50—C51	81.6 (5)
C2—N3—N4—C10	106.3 (5)	C45—N44—C50—C51	−155.4 (4)
N3—N4—C5—C6	52.0 (4)	N44—C50—C51—C52A	−86.4 (8)
C10—N4—C5—C6	−72.6 (4)	N44—C50—C51—C54B	72.6 (10)
N3—N4—C5—C7	−75.3 (4)	N44—C50—C51—C53A	151.8 (8)
C10—N4—C5—C7	160.1 (3)	N44—C50—C51—C52B	−52.1 (10)
C2—O1—C6—C5	35.1 (5)	N44—C50—C51—C54A	32.0 (9)
N4—C5—C6—O1	−61.2 (4)	N44—C50—C51—C53B	−168.8 (9)
C7—C5—C6—O1	63.5 (4)	C66—O61—C62—O75	−178.0 (4)
N4—C5—C7—C9	−60.6 (4)	C66—O61—C62—N63	1.6 (6)
C6—C5—C7—C9	176.0 (4)	O75—C62—N63—N64	178.0 (4)
N4—C5—C7—C8	175.9 (3)	O61—C62—N63—N64	−1.5 (6)
C6—C5—C7—C8	52.6 (5)	C62—N63—N64—C70	97.4 (4)
N3—N4—C10—C11	118.9 (4)	C62—N63—N64—C65	−28.1 (5)
C5—N4—C10—C11	−118.5 (4)	N63—N64—C65—C66	54.1 (4)
N4—C10—C11—C14	62.7 (4)	C70—N64—C65—C66	−70.2 (4)
N4—C10—C11—C13	−59.7 (4)	N63—N64—C65—C67	−72.5 (4)
N4—C10—C11—C12	−179.0 (3)	C70—N64—C65—C67	163.2 (3)
C26—O21—C22—O35	177.6 (4)	C62—O61—C66—C65	28.0 (6)
C26—O21—C22—N23	−6.0 (6)	N64—C65—C66—O61	−56.3 (4)
O35—C22—N23—N24	173.1 (4)	C67—C65—C66—O61	68.0 (4)
O21—C22—N23—N24	−3.2 (6)	N64—C65—C67—C69	−48.6 (5)
C22—N23—N24—C25	−21.1 (5)	C66—C65—C67—C69	−171.5 (4)
C22—N23—N24—C30	104.9 (5)	N64—C65—C67—C68	−172.3 (4)
N23—N24—C25—C26	50.9 (4)	C66—C65—C67—C68	64.8 (5)
C30—N24—C25—C26	−73.1 (4)	N63—N64—C70—C71	105.7 (4)
N23—N24—C25—C27	−75.6 (4)	C65—N64—C70—C71	−132.2 (4)
C30—N24—C25—C27	160.5 (3)	N64—C70—C71—C73	−61.7 (6)
C22—O21—C26—C25	37.7 (5)	N64—C70—C71—C72	176.7 (4)
N24—C25—C26—O21	−60.8 (4)	N64—C70—C71—C74	61.0 (6)
C27—C25—C26—O21	64.0 (4)	C86—O81—C82—O95	−175.7 (4)
N24—C25—C27—C29	−57.2 (4)	C86—O81—C82—N83	3.1 (6)
C26—C25—C27—C29	179.3 (4)	O95—C82—N83—N84	178.4 (4)
N24—C25—C27—C28	−179.8 (3)	O81—C82—N83—N84	−0.4 (6)
C26—C25—C27—C28	56.8 (5)	C82—N83—N84—C85	−31.3 (5)
N23—N24—C30—C31	120.1 (4)	C82—N83—N84—C90	93.7 (4)
C25—N24—C30—C31	−117.4 (4)	N83—N84—C85—C86	56.7 (4)
N24—C30—C31—C34	61.6 (5)	C90—N84—C85—C86	−66.5 (4)
N24—C30—C31—C32	179.8 (4)	N83—N84—C85—C87	−70.3 (4)
N24—C30—C31—C33	−59.2 (5)	C90—N84—C85—C87	166.5 (3)
C46—O41—C42—O55	−178.8 (4)	C82—O81—C86—C85	25.6 (6)
C46—O41—C42—N43	1.0 (6)	N84—C85—C86—O81	−55.8 (4)
O55—C42—N43—N44	174.1 (4)	C87—C85—C86—O81	68.9 (4)
O41—C42—N43—N44	−5.7 (6)	N84—C85—C87—C89	−54.8 (5)
C42—N43—N44—C45	−23.6 (5)	C86—C85—C87—C89	−177.8 (4)
C42—N43—N44—C50	102.9 (5)	N84—C85—C87—C88	−177.5 (4)
N43—N44—C45—C46	54.1 (4)	C86—C85—C87—C88	59.5 (5)
C50—N44—C45—C46	−70.5 (4)	N83—N84—C90—C91	91.5 (4)
N43—N44—C45—C47	−73.9 (4)	C85—N84—C90—C91	−146.5 (4)
C50—N44—C45—C47	161.6 (4)	N84—C90—C91—C93	−55.0 (6)
C42—O41—C46—C45	31.4 (5)	N84—C90—C91—C92	−179.4 (4)
N44—C45—C46—O41	−59.2 (5)	N84—C90—C91—C94	64.5 (5)
C47—C45—C46—O41	65.3 (5)		

### Hydrogen-bond geometry (Å, °)

**Table:**

*D*—H···*A*	*D*—H	H···*A*	*D*···*A*	*D*—H···*A*
N23—H23···O55^i^	0.78 (4)	2.16 (4)	2.937 (5)	177 (5)
N43—H43···O35^ii^	0.88 (4)	1.99 (4)	2.868 (5)	175 (3)
N63—H63···O95^iii^	0.84 (4)	2.13 (4)	2.949 (5)	163 (3)
N83—H83···O75^iv^	0.89 (4)	2.04 (4)	2.926 (4)	175 (4)

Symmetry codes: (i) −*x*+1, *y*−1/2, −*z*+1; (ii) −*x*+1, *y*+1/2, −*z*+1; (iii) *x*, *y*, *z*−1; (iv) *x*, *y*, *z*+1.
